# Tracing Human Diversity in South America's Southern Cone: Linguistic, Morphometric, and Genetic Perspectives

**DOI:** 10.1002/ajpa.70077

**Published:** 2025-06-28

**Authors:** Lumila Paula Menéndez, Matthias Urban

**Affiliations:** ^1^ Department Anthropology of the Americas University of Bonn Bonn Germany; ^2^ School of Anthropology University of Costa Rica San José Costa Rica; ^3^ Unit of Theoretical Biology, Department of Evolutionary Biology University of Vienna Vienna Austria; ^4^ Centre National de la Recherche Scientifique Laboratoire “Dynamique du Langage” (UMR 5596) Lyon France; ^5^ Université Lumière Lyon 2 Lyon France

**Keywords:** biological and cultural diversity, human evolution, late Holocene, pairwise matrix correlation, South America

## Abstract

**Objectives:**

Studying the relationship between biological and cultural diversity can lead to rich insights into human history. South America has been relatively neglected in this kind of work, even though it intriguingly exhibits unexpectedly high biological and cultural diversity. Here, we focus on a particularly understudied part of the continent, the Southern Cone, and examine linguistic, craniometric, and genetic variation across five groups: Selk'nam, Qawaskar, Mapuche, Kunza, and Qom.

**Materials and Methods:**

We retrieved craniometric and genetic data from public databases and coded linguistic data capturing variation in sound systems and grammatical structures specifically for this study. We calculated distance matrices (Mahalanobis, Jaccard, F_ST_) and compared them using partial Mantel, Procrustes analysis, and multidimensional scaling in R.

**Results:**

Selk'nam and Qawaskar exhibit the strongest linguistic and craniometric similarities, likely due to geographic proximity, while Mapuche and Qom are the most genetically similar, reflecting recent migrations. Consistent with global studies, we observed a statistically significant correlation between the relatively plastic cranial vault morphology and the quickly evolving linguistic variables. Genetic variability was moderately related to geography, while the weakest correlation was found between the temporal bone morphology and genetic variation.

**Discussion:**

Although this study is limited by a small sample size and requires further research validation with larger datasets, our findings highlight the importance of integrating multiple datasets to better understand the interplay between biological and cultural diversity in shaping human history. Our findings also indicate that structural linguistic data help reconstruct population history, particularly at recent and intermediate scales.

## 1 Introduction

In the isolation by distance model that is usually employed to describe the Out‐of‐Africa (OA) expansion of humans across continents (Betti et al. 2009; Cavalli‐Sforza 1994; Nielsen et al. 2017), genetic drift is the main evolutionary factor acting on human variation. Human diversity should decrease with increasing distance from Africa along the path of Pleistocene human expansions in this model. Therefore, Native Americans are expected to be the genetically least differentiated human populations worldwide, that is, the least heterogeneous when compared to human populations from other continents (Howells 1989; Relethford 2010; Smith 2011)—a prediction that is largely borne out (Kemp and Schurr 2010; Wang et al. 2007; but see Castro e Silva et al. 2022). However, despite South America being the last continent to be effectively occupied by humans at least ~15,500 years BP (Prates et al. 2020), morphological variation among ancient human populations has been described as extremely high (González‐José et al. 2001; Sardi et al. 2005). Likewise, linguistic diversity in South America (Campbell 2024) is often considered inconsistent with the recent human presence (Nichols 1990, 2024; though see Nettle 1997), pointing to a further, cultural, dimension in which human prehistory on the continent may have been particularly complex or marked by early and prolonged diversification to yield the observed patterns.

Genetically, South America is characterized by high genetic diversity in certain regions, which contrasts with relatively low diversity compared to other global populations (Arencibia et al. 2019; Castro e Silva et al. 2022). This genetic diversity reflects the continent's complex population history. All non‐Arctic Native American groups descend from an ancestral population that migrated from Asia to Beringia and subsequently split into a northern branch (NNA or ANC‐B) and a southern branch (SNA or ANC‐A) (Raghavan et al. 2014; Scheib et al. 2018). It remains debated whether these ancestral lineages were influenced by South Asian groups or solely East Asian populations (Campelo dos Santos et al. 2022; Menéndez 2024; Moreno‐Mayar et al. 2018). The SNA branch, represented in North America by Anzick‐1 (Clovis) ~12,000 years BP (SNA1) and Spirit Cave (Western Stemmed Tradition) ~11,000 years BP (SNA2), spread across North, Central, and South America and entered the latter in multiple pulses (Moreno‐Mayar et al. 2018; Posth et al. 2018; Willerslev and Meltzer 2021). A rapid initial colonization is suggested by mtDNA data, which reveal unique lineages across the Americas (Kitchen et al. 2008; Llamas et al. 2016; Tamm et al. 2007). In South America, three major branches of genomic ancestry emerged early after colonization, characterizing populations in the Andes, the Amazon, and the Southern Cone. Later population dispersal, such as one from present‐day California ~4000 years BP (Posth et al. 2018) or the late Holocene Tupi‐Guaraní expansion (Castro e Silva and Hünemeier 2023), along with other, often smaller and/or undocumented migrations, further (re)shaped human diversity in South America.

Studies analyzing dental and cranial data from ancient South American individuals across multiple populations have characterized them as highly diverse, sometimes exhibiting greater variation than expected under the isolation‐by‐distance model (González‐José et al. 2001; Hubbe et al. 2014, 2015; Lahr 1995; Perez et al. 2009; Pucciarelli et al. 2008; Relethford 2010; Sardi et al. 2005; Sutter 2005). In contrast to this expectation, morphological variation within South America equals the variation observed between populations from different continents (González‐José et al. 2001, 2008; Ponce de León et al. 2018; Sardi et al. 2005). Initially, this significant variation was interpreted as the result of multiple ancestral lineages shaping the history of South Americans (Neves and Pucciarelli 1991; von Cramon‐Taubadel et al. 2017). More recently, non‐random factors involving the interplay between morphology and environmental influences, such as adaptations to cold climates, high altitudes, and diverse diets, are receiving increasing consideration (de Azevedo et al. 2017; Menéndez 2018; Menéndez et al. 2014; Perez and Monteiro 2009; Perez et al. 2011).

Linguistically, the Americas remain one of the most diverse parts of the world, despite the loss of many languages since European colonization (Campbell 2024). Within the Americas, the diversity of South America exceeds that of North America as measured both by the number of distinct languages as well as by the number of singular linguistic lineages (“language families”). Within South America, in turn, the Amazonian lowlands are generally more diverse than the Andes and the Southern Cone, though the uneven demographic and cultural impact of European conquest may have contributed to accentuating the observed diversity cline. About 60% of South American lineages are isolates (Seifart and Hammarström 2017), that is, consist of only one extant member whose ties with related lineages were severed at a point of time that exceeds the time horizon that can be reached by historical linguistic methods (under ideal conditions ~8000 years). This indicates high rates of linguistic diversification and cladogenesis already in an early phase of human presence. The causes of this remain poorly understood. South American languages are also characterized by extreme heterogeneity concerning their evolved sound systems and grammars—a third parameter of linguistic diversity that contributes to the overall complexity of the linguistic situation in the continent (e.g., Campbell 2012).

These intriguing differences between different variables of biocultural diversity raise questions about their origin and causes in ways that may be central to understanding the human story on the continent. To tackle this problem, several studies concerned with South America or the Americas as a whole evaluate associations between genetic and linguistic diversity (Fagundes et al. 2002; Hunley et al. 2007; Ramallo et al. 2013; Urban and Barbieri 2020), morphology and geography (De Azevedo et al. 2011; González‐José et al. 2001), and morphology and environmental factors (Béguelin 2010; Bernal et al. 2010; Menéndez 2018; Menéndez et al. 2014; Perez et al. 2011). In contrast, few studies have explored the association between genetic and morphological diversity (Delgado et al. 2021; Perez et al. 2009; Strauss and Hubbe 2010).

While the association between morphometric and linguistic diversity has been explored in some regions (Reyes‐Centeno et al. 2017), no research has examined this relationship in the Americas since the empirically and methodologically problematic work of Greenberg et al. (1986; see also Bolnick et al. 2004). Greenberg's model has been widely rejected by linguists for relying on superficial lexical comparisons and neglecting regular sound correspondences. It grouped most Native American languages into a single “Amerind” family, collapsing over 100 established language families and isolates into one group—contradicting decades of rigorous linguistic research. His multilateral comparison approach also failed to distinguish inherited features from chance similarities or borrowing. Despite its rejection by linguists, the model has been influential in genetic studies, even though more robust classifications (e.g., Campbell 1997, 2024) better explain genetic variation—by up to 40% in some cases—highlighting the need for linguistically grounded interdisciplinary research (Bolnick et al. 2004).

More importantly, no studies have examined the association between craniometric, genetic, and linguistic variation using the same set of populations. Although previous research in North, Central, and South America has analyzed genetic variation in samples grouped by linguistic families, it has generally not applied statistical tests to formally assess the relationship between genetic and linguistic variation (e.g., Amorim et al. 2013; Castro e Silva et al. 2022; Hunley et al. 2007; Johnson and Lorenz 2006; Kemp et al. 2005; Ramallo et al. 2013; Salzano et al. 2005; Ward et al. 1993). Exceptions include the work by Barrantes et al. (1990), who found strong and significant correlations between genetic and linguistic variation among Central American groups, and Fagundes et al. (2002), who reported similar findings for South America. However, no morphological data was included in these analyses. This gap limits our understanding of how different lines of evidence—biological and cultural—reflect population history. Integrating morphometric, genetic, and linguistic data from the same individuals or groups offers a more comprehensive framework for reconstructing human population dynamics and historical relationships.

Interdisciplinary efforts to refine our understanding of South American prehistory have primarily focused on the Middle Andes and Amazonia and explored, for example, the Inka, Tiwanaku, and Tupi expansions (Castro e Silva and Hünemeier 2023; Delgado et al. 2021; Ramallo et al. 2013; Urban and Barbieri 2020). South America's Southern Cone has been much less thoroughly explored from an interdisciplinary angle. However, as the last continental region to be settled by humans, and with its *cul‐de‐sac* shape that makes it a natural endpoint for southward dispersals, the Southern Cone holds particular significance for understanding the population history of the Americas. While some groups of the Cone, such as the Mapuche, received limited amounts of cultural and demographic impact from the *foci* of cultural developments in the Central Andes, the same group shows direct genetic continuity with earliest populations (Arango‐Isaza et al. 2023). At the same time, diverse adaptations and lifestyles—such as hunting and gathering, horticulture, and agriculture—were interacting in complex ways as suggested, among other things, by linguistic evidence (e.g., de Carvalho 2018; Fernández Garay 1997, 2012a, 2012b; Golluscio 2009; Malvestitti 2005; Pache 2014; Tovar 1951; Viegas Barros 1996, 2005, 140–152; Viegas Barros 2014, 586; Urban 2018). However, the overall demographic dynamics remain poorly understood.

The aim of this study is to evaluate the relationship between linguistic, genetic, and craniometric diversity in a sample of five groups from southern South America to shed new light on the origins of linguistic and biological diversity in the Southern Cone, as well as the interrelations and interactions among human groups.

In doing so, we take advantage of the differential plasticity of different parts of the skull, which tracks biological affinities across different time scales and reflects the influence of random and non‐random factors to different extents. The cranial base, including the temporal bone—and especially the petrous portion—reflects population affinities on a deep temporal scale because these structures complete their growth early in life, making them more stable and less influenced by environmental factors, that is, more conserved over time (Harvati and Weaver 2006; Martínez‐Abadías et al. 2009; Reyes‐Centeno et al. 2016; Smith et al. 2007; Terhune et al. 2007). Conversely, the facial skeleton and mandible are influenced throughout ontogeny by non‐random factors such as diet (Katz et al. 2016; Noback and Harvati 2015; Paschetta et al. 2016; von Cramon‐Taubadel 2011) and climate (Hubbe et al. 2009; Evteev et al. 2024; Maddux et al. 2016; Noback et al. 2011). As a result, compared to the cranial base, the facial skeleton reflects changes driven by more recent events.

The cranial vault of *Homo sapiens* exhibits significant variation across and within populations—ranging from antero‐posteriorly elongated skulls to globular shapes. This variation is considered highly integrated and mainly influenced by brain growth (Anzelmo et al. 2013; Howells 1957; Lieberman 2011). While some studies argue that cranial vault variation aligns with random genetic evolution (Smith 2009), others suggest that parts of the cranial vault are influenced by non‐random factors, similar to the face (Harvati and Weaver 2006; Evteev et al. 2024; Paschetta et al. 2010; von Cramon‐Taubadel 2011). In light of such differing interpretations, based on heterogeneous datasets and analyses, the most parsimonious explanation is that cranial vault variation is population‐specific, with different regions of the cranial vault influenced by distinct factors. Therefore, as the cranial vault is shaped by both random and non‐random factors, it reflects changes occurring at an intermediate temporal scale—between the recent timing of the face and the deep timing of the cranial base.

Language, in contrast, follows a distinct mode of evolution that is characterized by higher rates of change (Bickel et al. 2024). This has made it difficult to meaningfully relate it to the genetic record in general. However, morphometric evidence relating to the cranial vault and facial morphology has the potential to track demographic processes also indexed by linguistic change and differentiation (Reyes‐Centeno et al. 2016), a potential that remains largely unexplored.

Here, our main objectives are (1) to assess the biological and cultural affinities among South American groups by calculating and comparing patterns of variation derived from linguistic, craniometric, and genetic distance matrices; and (2) to evaluate the multivariate relationships among these datasets for five groups. Concerning (1), we expect the strongest linguistic, craniometric, and genetic similarities to occur between geographically proximate populations, such as the Selknam and Qawaskar, while the greatest differences are anticipated between more distant populations, such as the Qom and Selknam. This expectation aligns with the null hypothesis that the geographically proximate populations share a more recent common ancestry and experience frequent contact and gene flow, leading to linguistic, craniometric, and genetic similarities. For objective (2), consistent with previous studies conducted on a global scale, we anticipate a stronger correlation between linguistic variation and facial variation given the rapid rate of change in the facial skeleton suggested by previous studies.

## 2 Materials and Methods

### 2.1 Samples

**FIGURE 1:**
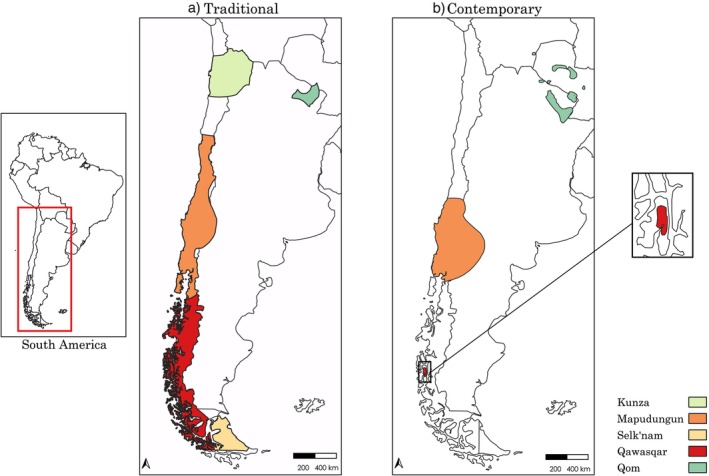
Map showing the geographical distribution of our study groups at the time of Indigenous‐European contact (“Traditional”) and in 2007 (“Contemporary”). “Contemporary” refers to the period during which data for the *Atlas of the World's Languages*, from which these data were extracted, were collected. The map was created in QGIS using a NaturalEarth base map and polygons derived from the *Atlas* (Asher and Moseley 2007). We acknowledge that the figure may not precisely reflect the geographic extent of Indigenous populations at these specific time points, but it serves as a model to approximate their geographic distribution over time.

We selected five groups from the Southern Cone for which linguistic, craniometric, and genetic data were available. As shown in Figure 1, the geographic distribution of those groups changed slightly over time (Asher and Moseley 2007). As the first step in the sample selection process, we evaluated the correspondence between samples in the largest craniometric dataset (Paschetta et al. 2017) with the availability of sufficiently detailed grammatical descriptions of native languages of the Southern Cone. After identifying groups for which matching craniometric and linguistic descriptions data are available, we searched for corresponding genetic data for these samples (Table 1). This further restricted potential target groups and yielded a small sample of five populations: the Selk'nam, Qawasqar, Qom, Mapuche, and Kunza (Table 1). While we acknowledge the limitation of working with such a small sample, we prioritized that the selected groups are reliably matched.

**TABLE 1:** Core target populations with precise matches across craniometric, linguistic, and genetic data used in the analysis.

Name used in this paper	Linguistic sample^a^	Craniometric sample	Genetic sample
Selk'nam	Selk'nam (Rojas Berscia 2014). Twentieth century.	Selk'nam (Pucciarelli et al. 2006). Late Holocene (*N* = 42)	Selk'nam (Lalueza Fox et al. 1997). Late Holocene, Nineteen century (*N* = 13)
Qawasqar	Qawasqar (Clairis 1985; Aguilera 2001; Dryer 2013a, 2013b). Twentieth century.	Alakaluf (Pucciarelli et al. 2006). Late Holocene (*N* = 24)	Qawasqar (de Saint Pierre et al. 2012). Contemporary individuals (*N* = 13)
Mapuche	Mapuche (Adelaar and Muysken 2004; Augusta 1996; Smeets 2008; Sadowsky et al. 2013). Eighteen–nineteen centuries.	Mapuche (Pucciarelli et al. 2006). Late Holocene (*N* = 28)	Mapuche Oeste (de Saint Pierre et al. 2012). Contemporary individuals (*N* = 53)
Kunza	Kunza (Adelaar and Muysken 2004; Peyró García 2005; Sáez Godoy 1974). Nineteen and twentieth century.	Coyo Oriental (Pucciarelli et al. 2006). Late Holocene (*N* = 16)	Atacameño (de Saint Pierre et al. 2012). Contemporary individuals (*N* = 28)
Qom/Toba	Qom (Messineo 2003; Lafone Quevedo 1896). Nineteen and twentieth century.	Noreste (Pucciarelli et al. 2006). Late Holocene (*N* = 19)	Combined data on Toba, from Argentinean provinces of Chaco and Formosa (Lewis Jr et al. 2005). Contemporary individuals (*N* = 56)
Total of samples analyzed	N/A	129	163

*Note:* Chronological information is provided for all three datasets, while sample sizes are specified for the craniometric and genetic data. Sample size is listed as not available (N/A) for the linguistic data, as it is difficult to determine; most authors rely on archival sources or do not report the number of subjects studied.^a^The abbreviations correspond to those used by H. M. Pucciarelli in his database. We present the English abbreviation followed by the Spanish version in brackets.

Historically, the Selk'nam lived in the central and northern parts of Isla Grande de Tierra del Fuego, while the Qawaskar inhabited the western and southwestern archipelagos (Borrero 2001; Chapman 2010; Gusinde 1982) (Figure 1). Traditionally, the Qawaskar, along with the Yaghan, have been classified as “Canoe Nomads” due to their maritime lifestyle and reliance on marine resources, such as seals and shellfish. In contrast, the Selk'nam, grouped with the Aonikenk and Haush, were known as “Foot Nomads” for their terrestrial subsistence strategies and dependence on land animals like *Lama guanicoe* (Borrero 2001; Chapman 2010; Gusinde 1982). Despite these lifestyle differences, genetic studies suggest 7000 years of shared ancestry in the Fuegian region, with some scholars proposing that the “Canoe Nomads” descended from the “Foot Nomads” (De la Fuente et al. 2015, De la Fuente et al. 2018; Nakatsuka et al. 2020; but see Balentine et al. 2023 for an alternative perspective). The Selk'nam language belongs to the Chonan family, which includes mainland groups like the Teushen and Tehuelche and insular groups from Isla Grande de Tierra del Fuego, such as the Haush and Selk'nam (Viegas Barros 2014). The Qawaskar language, spoken in Puerto Edén on Wellington Island and historically in broader areas around the Gulf of Penas (Clairis 1985; Figure 1), is part of the Alacalufan (or Kawesqar) linguistic family. This small family includes dialects that might represent distinct languages (Viegas Barros 2005).

The Qom inhabit the southern Chaco, and the Mapuche northern Patagonia (Figure 1), with the latter also present in parts of the Pampas, as a result of more recent migrations. The Qom (Toba) language belongs to the Guaicuruan language family, which is native to the Chaco region. Mapudungun, the Mapuche language, is spoken across central Chile (including, with clear dialectal differences, Chiloé Island) and parts of Argentina and has a relatively large number of speakers. Despite extensive documentation, there are no confirmed genealogical relationships.

Kunza (or Atacameño), finally, was the language of the people of the San Pedro de Atacama oasis, and possibly a much larger area (Figure 1). They entertained strong historical, cultural, and commercial ties with the Central Andes; this language, now considered dormant (Adelaar and Muysken 2004, 376), is also unrelated to other known native languages genealogically.

### 2.2 Data Acquisition

Craniometric data was obtained from the online database collected by Héctor Pucciarelli (Paschetta et al. 2017; Pucciarelli et al. 2006). This is the largest publicly available morphometric database for South American populations. The full dataset includes 30 craniofunctional variables, defined and recorded using the “craniofunctional method,” from 1607 individuals representing 93 South American groups (Pucciarelli 2008; Sardi 2017). The craniofunctional method, developed by Pucciarelli, is based on the Functional Matrix Hypothesis (Moss and Young 1960) and provides an alternative to classical craniometry by enabling the evaluation of factors influencing skull morphology. The variables are linear measurements obtained using different types of specialized calipers.

For this study in particular, we selected 129 individuals representing five South American groups (Pucciarelli 2008; Sardi 2017; Figure 1). From the 30 craniofunctional variables available, we chose subsets to evaluate morphological variation in specific cranial modules: the face, cranial vault, and temporal bone (Table 2). A total of 15 variables describes facial morphology, nine describe the neurocranium, and three describe the temporal bone (Table 2). These broader categories allow us to analytically distinguish different parts of the skull, which reflect ecological factors and evolutionary processes in distinct ways (Roseman 2016; Roseman and Weaver 2004; Smith et al. 2007; von Cramon‐Taubadel 2014) and to examine their relationships with genetic and linguistic variation separately.

**TABLE 2:** Craniometric variables used in this study.

Cranial module	Abbreviation^a^	Name, definition, and instrument used to measure it
Facial	FL (LF)	Facial length: Inner Prosthion‐Vomerbasio. Sliding (Poech type). Projected.
	FW (AF)	Facial width: Zygion‐Zygion. Spreading Caliper. Direct.
	FH (HF)	Facial height: Nasion‐Prosthion. Sliding (Poech type). Projected.
	OL (LO)	Optic length: Dacrion‐Superior orbital fissure. Orbitometer. Direct.
	OW (AO)	Optic width: Dacrion‐Ectoconquio. Vernier Caliper. Direct.
	OH (HO)	Optic height: Maximum height from the upper to the lower orbital borders, perpendicular to the horizontal axis of the orbit. Sliding (Poech type). Direct.
	RL (LR)	Respiratory length: Nasospinale‐Staphylion. Spreading Caliper. Direct.
	RW (AR)	Respiratory width: Left Alare‐right Alare. Vernier Caliper. Direct.
	RH (HR)	Respiratory height: Nasion‐Nasospinale. Sliding (Poech type). Projected.
	ML (LM)	Masticatory length: Distance from the zygomaxillare anterior to the posterior margin of the glenoid fossa. Sliding (Poech type). Projected.
	MW (AM)	Masticatory width: Distance from the anterior border of the sphenoid bone in the greater wing to the lowest point of the zygotemporal suture. Vernier Caliper. Projected.
	MH (HM)	Masticatory height: Distance from the stephanion to the lowest point of the zygotemporal suture. Sliding (Poech type). Projected.
	AL (LA)	Alveolar length: external Prosthionposterior alveolar border. Vernier Caliper. Direct.
	AW (AA)	Alveolar width: Left ectomolare‐right ectomolare. Vernier Caliper. Direct.
	AH (HA)	Alveolar height: Palatal depth on the palatine suture, measured by placing the lateral arms of the palatometer on the left and right ectomolare. Palatometer. Direct.
Neurocranium	ANL (LNA)	Anteroneural length: Glabella‐Bregma. Sliding (Poech type). Projected.
	ANW (ANA)	Anteroneural width: Pterion‐Pterion. Spreading Caliper. Direct.
	ANH (HNA)	Anteroneural height: Bregma‐Vomerbasio. Spreading Caliper. Direct.
	MNL (LNM)	Midneural length: Bregma‐Lambda. Sliding (Poech type). Projected.
	MNW (ANM)	Equal to AN (NW). Spreading Caliper. Direct.
	MNH (HNM)	Midneural height: Basion‐Bregma. Spreading Caliper. Direct.
	PNL (LNP)	Posteroneural length: Opisthion‐Opisthocranium. Sliding (Poech type). Projected.
	PNW (ANP)	Posteroneural width: Asterion‐Asterion. Spreading Caliper. Direct.
	PNH (HNP)	Posteroneural height: Lambda‐Opisthion. Sliding (Poech type). Projected.
Temporal bone	OTL (LOT)	Otic length: Distance from the external auditory meatus to the midpoint of the inner border of the petrous bone. Vernier Caliper. Direct.
	OTW (AOT)	Otic width: External auditory meatus width. Vernier Caliper. Direct.
	OTH (HOT)	Otic heigth: External auditory meatus height. Vernier Caliper. Direct.

^a^The abbreviations correspond to those used by H. M. Pucciarelli in his database. We present the English abbreviation followed by the Spanish version in brackets.

Our linguistic dataset assesses the basic organization of sound structure (phonology), word structure (morphology), sentence structure (syntax), and lexicon based on the identified primary descriptions. We survey 77 linguistic properties in these domains that are known to vary across the Americas, following Urban et al. (2019) (Table 3).

**TABLE 3:** Linguistic variables used in this study.

Area	Subarea	Feature
Phonology	Consonants	Is there a voicing contrast in stops?
		Are there phonemic glottalized/ejective consonants?
		Are there phonemic aspirated consonants?
		Are there phonemic uvulars?
		Is there a labiodental fricatives?
	Vowels	Is there a phonemic high central vowel?
		Is there phonemic vowel length?
		Is there phonemic vowel nasalization?
		Are there more than two phonemically relevant degrees of aperture?
		Are there more than three phonemically relevant degrees of aperture?
	Suprasegmental	Are there contrastive tones?
		Is there contrastive stress?
	Syllable Structure	Are there codas?
		Are there complex onsets?
Morphology	General	Is there a preference for prefixes in nominal inflectional morphology?
		Is there a preference for suffixes in nominal inflectional morphology?
		Is there a preference for prefixes in verbal inflectional morphology?
		Is there a preference for suffixes in verbal inflectional morphology?
		Is there productive reduplication?
	Transcategorical operations	Is there productive nominalizing morphology?
		Is there productive verbalizing morphology?
	Parts of speech	Is there a morphosyntactically definable class of adjectives?
	Nominal morphology	Are there possessive classes?
		Are there numeral classifiers?
		Are there noun classes/genders?
		Are there noun classifiers?
		Is there an inclusive/exclusive distinction in independent prounouns?
		Can inanimates be marked for plurality?
		Can human nouns be marked for plurality?
		Are there cases for other than core relations?
	Verbal morphology	Is there verbal person marking only for the A argument?
		Is there verbal person marking only for the P argument?
		Is there verbal person marking for the A or the P argument, but not both?
		Is there verbal person marking for both A and P arguments?
		Is tense a verbal category?
		Is aspect a verbal category?
		Are there directional affixes on verbs?
		Are there valency‐changing prefixes?
		Are there valency‐changing suffixes?
Syntax	Possession	Are possessive phrases dependent‐marked?
		Are possessive phrases head‐marked?
		Is there a verb ‘to have’ in predicative possession?
	Alignment	Does case marking in full NPs operate on a nominative‐accusative‐basis?
		Does case marking in full NPs operate on an ergative‐absolutive basis?
		Does case marking in full NPs operate on an tripartite basis?
		Does case marking in full NPs operate on an active‐inactive basis?
		Does verbal person marking operate on a nominative‐accusative‐basis?
		Does verbal person marking operate on an ergative‐absolutive‐basis?
		Does verbal person marking operate on an active‐stative‐basis?
		Does verbal person marking operate on a hierarchical basis?
		Does verbal person marking operate on more than one of the above systems?
	Word order	Is the dominant constituent order in intransitive clauses VS?
		Is the dominant constituent order in intransitive clauses SV?
		Is the dominant constituent order in transitive clauses VS?
		Is the dominant constituent order in transitive clauses VO?
		Is the dominant constituent order in transitive clauses OS?
		Is the dominant order in possessive phrases possessor‐possessed?
		Is the dominant order in possessive phrases possessed‐possessor?
		Is the dominant order in NPs adjective‐noun?
		Is the dominant order in NPs noun‐adjective?
		Is the dominant order in NPs demonstrative‐noun?
		Is the dominant order in NPs noun‐demonstrative?
		Is the dominant order in NPs numeral‐noun?
		Is the dominant order in NPs noun‐numeral?
		Is the element indicating clausal negation preceding the verb (root)?
	Complex constructions	Are there structural similarities between nominalization and relativization?
		Is there a system of switch‐reference?
		Is there a morphological passive?
Lexicon	Morpheme Canon	Are nominal roots predominantly monosyllabic?
		Are nominal roots predominantly disyllabic?
		Are verbal roots predominantly monosyllabic?
		Are verbal roots predominantly disyllabic?
	Numerals	Is some part of the numeral system organized on a quinary basis?
		Is some part of the numeral system organized on a decimal basis?
		Is some part of the numeral system organized on a vigesimal basis?
	Basic orientation	Is the underived word for ‘smoke’ a verb?
		Is the underived color word for ‘black’ a verb?

Finally, we obtained genetic data from a published study in which genetic distances were calculated for 18 South American groups (Evteev et al. 2024). These genetic distances were derived from the frequencies of the five main mtDNA haplogroups (Table 4), using the R package FinePop v1.5.1 (Kitada et al. 2007). The mtDNA data were sourced from previously published studies (Table 1). The resulting genetic distances are represented by the widely used Fixation Index (F_ST_) between groups, as described by Weir and Cockerham (1984). Geographic data were obtained from the geographic coordinates that reflect the recent geographical distribution of the sampled languages from Glottolog 5.1 (Hammarström et al. 2024).

**TABLE 4:** Percentage of mtDNA haplogroups in each studied group.

	A	B	C	D	References
Selk'nam	0	0	46.1	46.1	Lalueza et al. (1997)
Qawasqar	0	0	7.7	0	De Saint Pierre et al. (2012)
Mapuche	0	26.3	24.9	21.2	De Saint Pierre et al. (2012)
Kunza	0	0	3.6	3.6	De Saint Pierre et al. (2012)
Qom/Toba	20.1	40.6	5.2	30.6	Lewis Jr et al. (2005)

### 2.3 Statistical Analysis

We calculated F_ST_ distances for genetic data (Table S1), Mahalanobis distances for craniometric data (Table S2), Jaccard distances for linguistic data (Table S3), and geographical distances between the five populations according to the Vincenty formula (Table S4). All distance matrices used in this study are available in the Supporting Information.

Jaccard distance is the ratio of the size of the intersection to the size of the union of two datasets (Jaccard 1901). It is particularly well‐suited for binary data (presence/absence matrices) and is unaffected by missing values, as it only considers the presence or absence of elements. The Jaccard distance ranges from 0 to 1, where a distance of 0 indicates that the compared sets are identical, and a distance of 1 indicates that the sets are entirely dissimilar.

Mahalanobis distance is a multivariate metric that measures the distance of a data point from the mean of a distribution while accounting for correlations between variables and differences in their scales (Mahalanobis 1936). It is particularly valuable in multivariate analyses and is widely used in biological anthropology because it incorporates the covariance structure of the data. This makes it sensitive to variable interdependencies when analyzing morphological differences (Sneath and Sokal 1973). A larger Mahalanobis distance between two populations indicates greater morphological or genetic differentiation, while a smaller distance reflects stronger similarities between the compared groups.

Genetic distances are represented by the Fixation Index (F_ST_), a measure commonly used in population genetics to quantify genetic differentiation or variation between populations (Weir and Cockerham 1984). F_ST_ is based on allele frequencies and compares genetic diversity within populations to the total genetic diversity across all populations. An F_ST_ value of 0 indicates that populations are genetically identical with no differentiation, while an FST value of 1 indicates that the populations are completely distinct from each other.

**FIGURE 2:**
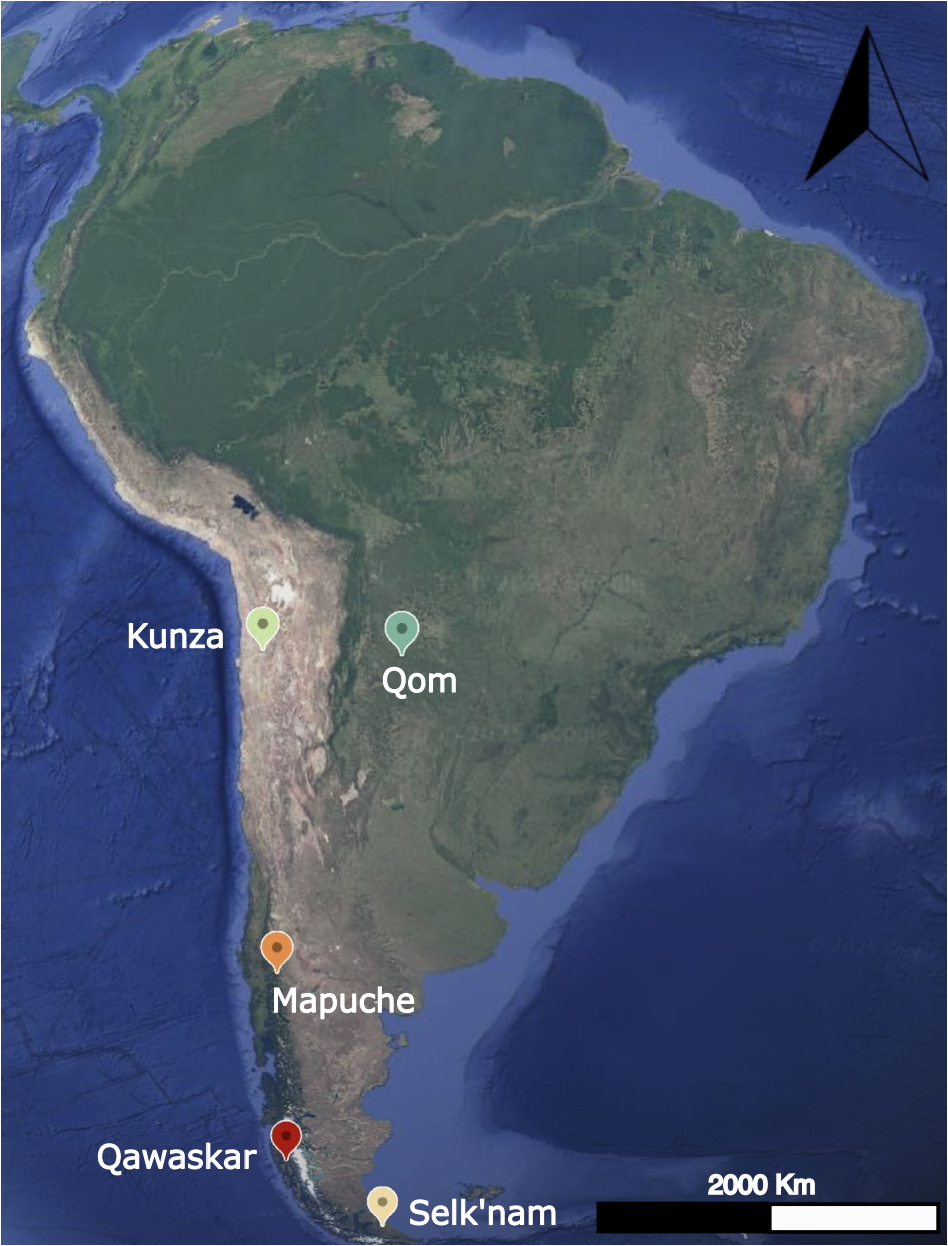
Map showing the location of the studied samples. The map was created using Google Earth (May, 2025).

Geographic distances were calculated using the Vincenty formula, as implemented in the geosphere R package, based on the locations shown in Figure 2. These locations corresponded to the centroids of the areas presented in Figure 1. The Vincenty formula is a highly accurate method for determining geographic distances between two points on the Earth's surface. Unlike methods based on a spherical model, the Vincenty formula uses an ellipsoidal model of the Earth, making it more precise for most practical applications.

To assess the correlations between the linguistic, craniometric, genetic, and geographic distances (Tables S1–S4), we used two widely applied matrix comparison methods: partial Mantel analysis and Procrustes analysis as implemented in the vegan R package. These permutation‐based methods are well‐suited for small datasets. Additionally, they are robust, easy to interpret, and widely used, which facilitates comparisons across studies.

The partial Mantel test has been widely used in anthropology to assess the relationship between two distance matrices (e.g., genetic, craniometric, linguistic, environmental) while controlling for the effects of a third distance matrix (e.g., geographic or genetic distances) (Evteev et al. 2017; Irish et al. 2020; Katz et al. 2016; Reyes‐Centeno et al. 2016, 2017; von Cramon‐Taubadel 2016). However, its application has been criticized because it assumes linear relationships among the distance matrices, an assumption that may not hold for all data types due to the complex structures of distance matrices (e.g., autocorrelation, non‐independence) (Guillot and Rousset 2013; Legendre and Fortin 2010). To address these limitations, we complemented the partial Mantel test, here based on Pearson's product–moment correlation, with Procrustes analysis. Due to the small dataset, alternative approaches such as distance‐based redundancy analysis (db‐RDA) were not applicable. In the partial Mantel tests, we included a third matrix (geographic distance) as a covariate to correct for spatial autocorrelation.

Procrustes analysis assesses the similarity between two datasets by minimizing differences in their geometric configurations. One key advantage is the ability to compare multidimensional datasets derived from different types of measurements (e.g., genetic and linguistic data). By standardizing and transforming the datasets, Procrustes analysis reduces the effects of initial differences in scale, orientation, or position. In addition to its widespread use in geometric morphometrics, Procrustes analysis has been applied to evaluate associations between genetic variation and geographic distribution (Wang et al. 2010), as well as among cranial, genetic, and geographic variation (Bernal et al. 2010).

We performed both partial Mantel and Procrustes analyses using the vegan R package with 119 permutations in both cases (the number of possible unique permutations is determined by the dataset size. For 5 items, there are 5! = 120 possible permutations, but one corresponds to the observed data. This leaves 120–1 = 119 permutations for testing against the null hypothesis). Permutation tests incorporate the data structure into *p*‐value calculations by generating a null distribution of the test statistic through random shuffling of data points. The observed test statistic is then compared to this null distribution, and the *p* value is computed based on the proportion of permuted test statistics that are as extreme or more extreme than the observed value.

Finally, to explore and visualize the relationships among craniometric, genetic, linguistic, and geographic distances, we first conducted a classical multidimensional scaling (MDS) analysis separately for each dataset using base R. Classical MDS is a dimension‐reduction technique that projects a distance matrix into a lower‐dimensional Euclidean space, preserving the relative pairwise dissimilarities among items as faithfully as possible. Each distance matrix—derived from cranial measurements, mtDNA data, structural linguistic features, and pairwise geographic coordinates—was reduced to two dimensions to facilitate visualization in two‐dimensional space. We then applied Procrustes superimposition using the vegan package in R to align the craniometric, genetic, and linguistic MDS configurations onto the geographic configuration, which was used as a reference. This method minimizes the squared distances between matched points through scaling, rotation, and translation, allowing for a comparative assessment of spatial concordance across datasets. The resulting aligned configurations were visualized using the ggplot2 package in R, with arrows representing the transformations from geographic positions to each aligned dataset.

## 3 Results

### 3.1 Calculation of Linguistic, Craniometric, and Genetic Distance Matrices in the Studied Groups

The obtained linguistic, craniometric, and genetic distances vary. Selk'nam and Qawaskar, that is, the geographically closest groups, are the most similar groups linguistically (*d* = 0.444), while the Qawaskar and Qom are the most dissimilar (*d* = 0.769). Morphometric distances show that the Qom and Mapuche are the most dissimilar groups in the sample (*D* ^2^ = 26.436), while the Selk'nam and Qawaskar again are the most similar (*D* ^2^ = 4.677), aligning with the linguistic data. In contrast, genetic distances reveal that the Mapuche and Qom are the most similar groups (FST = 0.002), which is opposite to the morphometric results. The Selk'nam and Kunza, in contrast, are the most genetically dissimilar (FST = 0.704).

### 3.2 Examining Multivariate Relationships Among the Morphometric, Genetic, and Linguistic Data

According to Partial Mantel analysis, the only statistically significant association is between linguistic variation and the morphology of the cranial vault and the temporal bone (Table 5). There are further notable, though not statistically significant, associations between linguistic and cranial data; however, facial variation is clearly unrelated and appears to attenuate the overall relationship (Table 5). The weakest association is observed between cranial and genetic variation, while the relationship between genetic and linguistic variation is intermediate.

**TABLE 5:** Results of the partial Mantel analysis.

	*r*	*p*
Linguistic/genetic	0.058	0.408
Genetic/linguistic	0.058	0.433
Cranial/linguistic	0.393	0.083
Linguistic/cranial	0.393	0.083
Cranial/genetic	−0.464	0.150
Genetic/cranial	−0.464	0.092
Facial/linguistic	0.0877	0.491
Facial/genetic	−0.161	0.333
Neurocranium/linguistic	**0.818**	**0.008**
Neurocranium/genetic	0.214	0.250
Temporal/linguistic	0.504	0.050
Temporal/genetic	−0.647	0.058

*Note:* Statistically significant values are indicated in bold.

The results of the Procrustes analysis are broadly similar. The strongest statistically significant association is between cranial vault and linguistic variation, followed by genetic and geographic variation (Table 6). Other notable associations include those between linguistic and cranial variation and between linguistic and geographic variation (Table 6).

**TABLE 6:** Results of the Procrustes analysis.

	m12 Squared	*p*
Linguistic/genetic	0.333	0.1
Linguistic/cranial	**0.201**	**0.066**
Cranial/genetic	0.599	0.491
Cranial/geographic	0.556	0.508
Linguistic/geographic	0.277	0.083
Genetic/geographic	**0.147**	**0.041**
Facial/linguistic	0.268	0.358
Facial/genetic	0.566	0.408
Facial/geographic	0.592	0.550
Neurocranium/linguistic	**0.144**	**0.025**
Neurocranium/genetic	0.278	0.108
Neurocranium/geographic	0.419	0.200
Temporal/linguistic	0.466	0.108
Temporal/genetic	0.891	0.550
Temporal/geographic	0.419	0.200

*Note:* Statistically significant values are indicated in bold.

**FIGURE 3:**
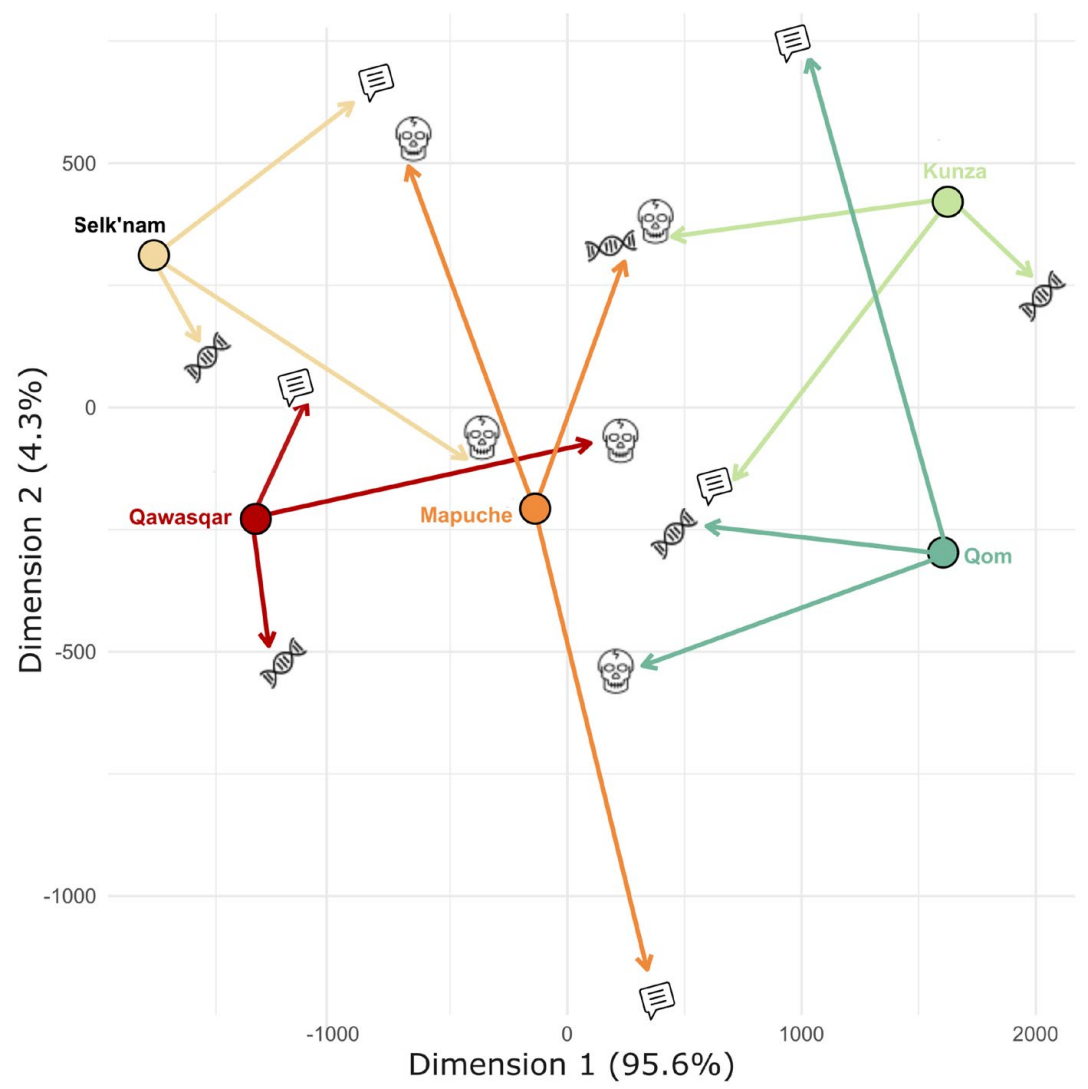
Multidimensional scaling analysis and Procrustes superimposition plot comparing the geographical distribution of the studied groups to craniometric (skull), genetic (DNA helix), and linguistic (speech bubble) distance matrices.

The Procrustes superimposition plot displays the alignment of the resulting MDS craniometric, genetic, and linguistic distance configurations onto a shared geographic reference (Figure 3). Each population is represented by four icons: a filled circle indicating its geographic position, and figurative icons indicating its position in each of the three aligned datasets. Arrows connect the geographic position to the corresponding position in each dataset, visualizing the magnitude and direction of the transformation. Linguistic configurations cluster near the geographic reference for Selk'nam and Qawaskar, with shorter arrows indicating stronger spatial correspondence. In contrast, craniometric and genetic configurations cluster more closely with the geographic reference in Qom, Mapuche, and Kunza. Overall, when using the geographic configuration as a reference, the linguistic dataset shows the greater divergence, evidenced by longer arrows and more dispersed points, followed by the craniometric configuration, while the genetic dataset remains the most spatially aligned.

## 4 Discussion

### 4.1 Assessing Affinities Among the South American Groups Studied: Principal Patterns of Variation

Based on our data, we found that the Selk'nam and Qawaskar exhibit the strongest similarities in linguistic, genetic, and morphometric properties, while the Mapuche and Qom are also showing a high degree of genetic similarity. These differences reflect the varying influence of random and non‐random factors across the datasets as well as sample‐specific characteristics, which we discuss below. Overall, our results support our initial hypothesis that geographically proximate populations tend to exhibit greater similarities.

The strong similarities in language and morphology between the Selk'nam and Qawaskar may be attributed to geographic, biological, and ecological factors. These groups are the geographically closest in our sample (676 km), both inhabiting southern Patagonia. As reviewed in Section 2.1, subsistence preferences between “Foot Nomads” with which the Selk'nam have been associated and “Canoe Nomads” like the Qawasqar conceal genetic continuity. In addition, mating appears to have been frequent where groups were living in proximity to one another (Bridges 1886; Nakatsuka et al. 2020) (in fact, the ethnic group known as “Dawsonians” might represent an admixed group of Selk'nam and Qawaskar people living at the mission on Dawson Island; Aspillaga and Ocampo 1996; Turbon et al. 2017). Genetic studies describe the groups inhabiting the Tierra del Fuego region as having lower genetic variation than other South American populations—evidenced by the presence of only two of the four mtDNA lineages described in South America (Crespo et al. 2018; De la Fuente et al. 2015; Lalueza Fox et al. 1996, 1997; Postillone et al. 2020; de Saint Pierre et al. 2012). While morphometric studies reveal significant morphological heterogeneity also among Fuegians (Turbon et al. 2017; González‐José et al. 2001; Alfonso‐Durruty et al. 2017), previous craniometric studies are consistent with ours in indicating close affinities between Qawaskar and Selk'nam groups, setting them apart from the Yaghan (González‐José et al. 2001, González‐José et al. 2002; Lalueza Fox et al. 1996; but see Varela et al. 1993). These differences have been interpreted as resulting from both a shared ancestry and adaptations to the harsh environmental conditions of the region (De la Fuente et al. 2015; González‐José et al. 2002; Hernández et al. 1997; Lahr 1995; Nakatsuka et al. 2020; Perez et al. 2007).

The Qom and Mapuche exhibit the strongest genetic similarities among the groups we studied. The Qom are a lowland group from the southern Chaco, while the Mapuche historically inhabited northern Patagonia, later expanding to the Pampas. Recent genetic studies on the Mapuche suggest that their genetic profile reflects an ancient ancestry linked to early migration waves (SNA2), followed by prolonged isolation before recent gene flow from the Central Andes (Arango‐Isaza et al. 2023; Capodiferro et al. 2021). In fact, mtDNA affinities between Chaco and Andean populations have led some scholars to suggest a shared evolutionary history (Cabana et al. 2006; Russo et al. 2018). This is consistent with previous genetic studies, which have reported close biological affinities between the Qom and Mapuche (Cabana et al. 2006; Demarchi and García Ministro 2008; Sevini et al. 2013) despite the fact that they are not geographic neighbors. There are also recent southeastward migrations of the Qom to Neuquén in northern Patagonia (Clairis 1985; Sala et al. 2019). This region has been inhabited by the Mapuche for centuries, if not millennia. Given that our mtDNA data include recent individuals, such migrations might explain the strong genetic affinities observed in our results.

The most dissimilar groups vary depending on the dataset, but they share one feature: they are separated by large geographic distances, consistent with our expectations. Qawaskar and Qom exhibited the greatest linguistic differences. The largest cranial differences were observed between the Qom and Mapuche, likely reflecting their distant common ancestry and lack of contact prior to European arrival. Previous craniometric and mtDNA studies, which show that Chaco samples differ significantly from those of Patagonia (de Saint Pierre et al. 2012; Perez et al. 2007), are consistent with the “Pampas Araucanization” (Mandrini 1998; Métraux 1946) brought about by dispersals of Mapudungun speakers with associated Mapuche ancestry. The most pronounced genetic differences were found between the Selk'nam and Kunza, likely due to their distant geographic locations (3409 km), the limited interaction of people of the Atacama area with groups outside the Andean region, and a distant common ancestor. The Kunza and the Selk'nam originated from different ancestral South America branches, that is, Andes/Amazon as opposed to the Southern Cone (Posth et al. 2018).

### 4.2 Evaluating the Correlations in Linguistic, Morphometric, and Genetic Data Matrices: Interpretations and Insights

Overall, the results demonstrate a significant association between linguistic and cranial diversity—specifically neurocranial diversity—in South American groups, as well as between genetic and geographic variation. In contrast, the associations between genetic variation and both linguistic and cranial diversity are weaker and not statistically significant.

The significant association we found between human genetic variation and geography aligns with previous studies demonstrating that genetic differentiation among populations often correlates with geographic distance. Similarly, for ancient human populations, genetic studies have consistently highlighted the impact of geography on population structure. Previous studies of ancient populations in the Americas have shown that genetic and craniometric differentiation often follows geographic gradients, reflecting the effects of migration and settlement across diverse landscapes (González‐José et al. 2008; Moreno‐Mayar et al. 2018; Posth et al. 2018). This relationship reflects isolation by distance, where geographically closer populations tend to share more genetic similarities due to higher rates of gene flow, while populations separated by greater distances experience reduced gene flow and increased genetic differentiation (Wright 1943). It also aligns with research supporting genetic drift as a primary factor shaping genetic variation (Elhassan et al. 2014; Nei et al. 1995; Reyes‐Centeno et al. 2015).

Our findings concerning the association between cranial and linguistic diversity are partially consistent with those of Reyes‐Centeno et al. (2016), who suggested, on the basis of craniometric and linguistic data from other parts of the world, that the whole cranium and the face show the strongest association with variation in vocabulary, followed by the cranial vault (or neurocranium) and the temporal bone.

The absence of an association between language diversity and facial morphology in our study contradicts our initial expectations. One possible explanation is the strong influence of ecological pressures on facial morphology among South American populations, a pattern highlighted in several previous studies (de Azevedo et al. 2017; Menéndez 2018; Menéndez et al. 2014; Perez and Monteiro 2009; Perez et al. 2011). Such strong ecological influences may obscure other signals of population history. Another potentially relevant factor is the difference in linguistic variables used: while Reyes‐Centeno and collaborators assessed linguistic population structure using lexical data (i.e., word lists), our study focused on structural linguistic features. These different aspects of language may evolve at different rates, although this remains an open question (e.g., Greenhill et al. 2017).

Like Reyes‐Centeno et al. (2016), we observed that the temporal bone does not exhibit a strong association with linguistic diversity. If the temporal bone reflects deeper evolutionary history, this may not be captured in linguistic comparisons as suggested by Reyes‐Centeno et al. (2016). They also suggested that changes in the cranial vault, like those in the face, reflect recent evolutionary history. Here we consider that the cranial vault reflects both evolutionary history and a moderate influence of ecological factors, such as climate and diet (Evteev et al. 2024; Harvati and Weaver 2006; Paschetta et al. 2010). Diet is of course related to subsistence preferences and associated cultural practices; therefore, it may be more closely related to other aspects of culture like language, which is considered a salient marker of cultural identity in many cultures.

## 5 Final Considerations

To our knowledge, this is the first study to evaluate the association between linguistic, craniometric, and genetic variation by matching and analyzing datasets from the same human groups in South America. Our analysis revealed significant correlations between linguistic and cranial diversity, as well as between geographic and genetic variation. These findings reinforce the proposition that linguistic data, including structural features, can help reconstruct population history, particularly at recent and intermediate scales. Since the cranial vault showed the strongest association with language, we propose that the recent history of populations should be understood broadly, encompassing not only random factors (e.g., genetic drift) but also non‐random ones (e.g., climate, diet). Overall, our findings underscore the importance of integrating multiple datasets to better understand the complex interplay between biological and cultural diversity in shaping human history.

Our findings are based on a small sample, which limits the robustness and generalizability of the results. They may not be representative of broader populations or other groups, and they may not generalize to other South American populations or to similar datasets. Additionally, with only five groups, the analyses have low statistical power and a higher likelihood of Type II errors (i.e., failing to reject a false null hypothesis). Therefore, relevant relationships or patterns may go unnoticed. Despite this, we found significant associations between cranial vault and linguistic diversity, and we obtained consistent results from partial Mantel and Procrustes analyses, which suggest that our results are at least robust against conceptually different methodological approaches.

## Author Contributions

**Lumila Paula Menéndez:** conceptualization, methodology, investigation, formal analysis, funding acquisition, visualization, writing – review and editing, writing – original draft. **Matthias Urban:** conceptualization, writing – original draft, writing – review and editing, methodology, formal analysis, funding acquisition, investigation, data curation.

## Conflicts of Interest

The authors declare no conflicts of interest.

## Supporting information


**Table S1.** Fst distances based on genetic data.
**Table S2.** Mahalanobis distances based on craniometric data.
**Table S3.** Jaccard distances based on linguistic data.
**Table S4.** Geographical distances in kilometers.

## Data Availability

The data that supports the findings of this study are available in the supplementary material of this article.
